# Effects of *Rhodotorula mucilaginosa* on Growth, Antioxidant, and Immune Function, and Toll/Imd and JAK-STAT Signaling Pathways in Red Claw Crayfish (*Cherax quadricanatus*)

**DOI:** 10.1155/anu/4904293

**Published:** 2025-07-30

**Authors:** Qin Zhang, Liuqing Meng, Jiqing Li, Luoqing Li, Qinghui Zeng, Rui Wang, Dapeng Wang, Tong Tong, Yongqiang Liu, Huizan Yang

**Affiliations:** ^1^Guangxi Key Laboratory for Polysaccharide Materials and Modifications, Guangxi Marine Microbial Resources Industrialization Engineering Technology Research Center, School of Marine Sciences and Biotechnology, Guangxi Minzu University, 158 University Road 530008, Nanning, China; ^2^Guangxi Key Laboratory for Aquatic Genetic Breeding and Healthy Aquaculture, Guangxi Academy of Fishery Sciences, 8 Qingshan Road, Nanning 530021, China

**Keywords:** crustaceans, feed additive, hepatopancreas, probiotics, signal pathway activation

## Abstract

In aquaculture, the use of probiotics to improve growth, immunity, and stress resistance in crustaceans has gained increasing attention. This study examined the effects of incorporating different levels of *Rhodotorula mucilaginosa* (0.0, 0.1, 1.0, and 10.0 g/kg) into the diet on growth performance, antioxidant capacity (AOC), immune function, Toll/Imd, and JAK-STAT signaling pathways in red claw crayfish (*Cherax quadricanatus*). The investigation was conducted through a 56-day feeding trial. The main results are as follows: Compared with the control group (0.0 g/kg), different *R. mucilaginosa* levels significantly increased (*p* < 0.05) the specific growth rate (SGR) and weight gain rate (WGR) of red claw crayfish, significantly increased (*p* < 0.05) the activities of superoxide dismutase (SOD), catalase (CAT), glutathione peroxidase (GPX), glutathione S-transferase (GST), total AOC (T-AOC), and acid phosphatase (ACP) in hepatopancreas of red claw crayfish, and significantly upregulated (*p* < 0.05) the relative expression levels of tumor necrosis factor receptor-associated protein 6, akirin, immunodeficiency homolog, interferon regulatory factor 4, Toll-like receptor (TLR) 6, TLR 2, Janus kinase, signal transducer activator of transcription, tumor necrosis factor α, interleukin-1β, transforming growth factor-β1 genes in the hepatopancreas of red claw crayfish. In conclusion, *R. mucilaginosa* significantly enhanced red claw crayfish's growth, AOC, and immune function, and activated the Toll/Imd and JAK-STAT signaling pathways. In this experimental context, the ideal addition level of *R. mucilaginosa* is 1.0 g/kg.

## 1. Introduction

Red claw crayfish (*Cherax quadricarinatus*) is an aquaculture species with substantial economic importance. It demonstrates fast growth along with robust environmental adaptability, capable of withstanding low oxygen conditions, a broad spectrum of pH levels, variations in temperature, and even elevated ammonia concentrations [1, 2]. The red claw crayfish is widely farmed in various provinces stretching from Hainan to Jiangsu, Fujian, and Guangdong in China, and it is estimated that the market value will climb to $9.3 billion by 2024 [3]. With the expansion of farming scale, high-density aquaculture practices have become increasingly prevalent. However, under such intensive farming conditions, red claw crayfish experience significantly increased survival stress and become susceptible to various pathogens [4]. Particularly in high-density recirculating aquaculture systems, where water exchange is limited, pathogens can spread rapidly once introduced, often causing substantial economic losses [5]. This phenomenon has become a major bottleneck restricting the sustainable development of the red claw crayfish farming industry.

To address this challenge, antibiotics have traditionally been the primary approach for disease control [6]. Antibiotics can rapidly and effectively eliminate bacteria, thereby controlling disease outbreaks. However, prolonged antibiotic use may result in drug resistance and environmental contamination, negatively impacting both organisms and ecosystems [7]. The rising public concern over food safety and environmental protection has made the shortcomings of this control measure increasingly apparent. In response to these significant risks, researchers have begun exploring alternative approaches, with probiotics emerging as a focal point of investigation [8]. Probiotics, which serve as an alternative to antibiotics, improve water quality and enhance host immunity, have garnered considerable attention. They not only inhibit the growth and reproduction of pathogenic bacteria through competitive exclusion [9] but also promote nutrient absorption, thereby fostering the growth and development of aquatic organisms and improving feed efficiency [10]. These characteristics demonstrate the broad application prospects of probiotics in healthy aquaculture of red claw crayfish.

*Rhodotorula mucilaginosa* has drawn significant interest because of its exceptional adaptability and the wealth of bioactive substances it contains. It is capable of flourishing in a wide range of environments, from typical settings to extreme conditions like deep-sea hydrothermal vents and Arctic ice sheets [11]. This organism is abundant in natural carotenoids, lipids, and a variety of enzymes [12]. Research has shown that the carotenoids synthesized by *R. mucilaginosa*, such as β-carotene, torulene, and torularhodin, exhibit substantial antioxidant capabilities. These compounds efficiently eliminate free radicals and reduce the damage caused by oxidative stress to aquatic cells [13]. Additionally, the polyunsaturated fatty acids derived from *R. mucilaginosa* exhibit anti-inflammatory effects, contributing to the overall health of aquatic organisms [14]. In the past few years, significant advancements have been achieved in utilizing yeast within the aquaculture industry. Studies suggest that red yeast can function as a dietary supplement, improving both the growth and immune response of Nile tilapia (*Oreochromis niloticus*) [15] and koi carp (*Cyprinus carpio var. koi*) [16]. Furthermore, the marine red yeast demonstrates suppressive effects on Vibrio species, which enhances the survival probability of whiteleg shrimp (*Litopenaeus vannamei*) [17]. With ongoing advancements in research, the possible uses of red yeast in aquaculture are anticipated to increase considerably.

To date, there has been relatively little research examining the impacts of *R. mucilaginosa* supplementation on growth, antioxidant capability, immune response, and the related signaling pathways in red-clawed crayfish. As a result, the underlying mechanisms remain largely unknown. Therefore, this study selected an optimal strain of *R. mucilaginosa* as a feed additive for red claw crayfish to investigate its beneficial impacts on growth, antioxidant capacity (AOC), immune function, Toll/Imd, and JAK-STAT signaling pathways. This study seeks to explore how feed probiotics affect the innate immune response in red claw crayfish, thereby offering a scientific basis for the utilization and development of *R. mucilaginosa* in aquaculture feed.

## 2. Materials and Methods

### 2.1. Experimental Diets

The freeze-dried powder of *R. mucilaginosa*, supplied by Guangzhou Xinhaili Biotechnology Co., Ltd. in Guangzhou, China, contained over 1 × 10^10^ cells per gram. Based on wet weight, the nutritional profile of *R. mucilaginosa* was as follows: it comprised 81.17% moisture, 9.27% crude protein, 4.45% crude lipid, 4.2% total triglycerides, 1.3% β-glucan, 1.4 mg/kg of β-carotene, 1.0 mg/kg of astaxanthin, and 172 mg/kg of vitamin E. The *R. mucilaginosa* was preserved at −20°C in a refrigerator.

The safety assessment of *R. mucilaginosa* was conducted following the research method of Kang et al. [18]. Healthy juvenile crayfish (purchased from the South Propagation Base of Guangxi Academy of Fishery Sciences in Nanning, China; the initial body weight was 1.10 ± 0.09 g, and the initial body length was 3.08 ± 0.12 cm) were randomly divided into two groups (treatment group and control group), each comprising three replicates. A total of six aquaculture tanks (0.6 × 0.6 × 0.6 m) were utilized, with 10 crayfish housed in each tank. Water conditions were maintained as follows: water temperature at 26–28°C, dissolved oxygen at 4.7–6.2 mg/L, ammonia nitrogen at 0–0.25 ppm, nitrite at 0.01 ppm, nitrate at 0–10 ppm, pH at 7.6–7.8, and natural light (~10 h light: 14 h dark). In the treatment group, *R. mucilaginosa* was added to maintain a bacterial concentration of 1 × 10^8^ CFU/mL in aquaculture water, while no bacteria were added to the control group. One-third of the water volume was renewed daily, bottom feces were removed, 1 × 10^8^ CFU/mL *R. mucilaginosa* was supplemented (treatment group), and a basal diet was provided. The feeding behavior and survival of juvenile crayfish were observed for 7 days, with survival numbers recorded daily. By the conclusion of the cultivation period, all red claw crayfish demonstrated excellent growth and vitality, with no noticeable decline in feeding activity. Both the treatment and control groups achieved a survival rate of 100%.

All feed materials were of animal food grade and manufactured by Guangdong Hengxing Feed Industry Co., Ltd., located in Zhanjiang, China. Based on prior research findings [19] and safety assessments (1 × 10^8^ CFU/mL), experimental diets were formulated with varying concentrations of *R. mucilaginosa*. To prepare the feed, varying quantities of freeze-dried *R. mucilaginosa* powder were incorporated into the basal feed and mixed uniformly to attain final concentrations of 0, 0.1, 1.0, and 10.0 g/kg. Initially, the basal feed was pulverized to a 60-mesh size using a hammer mill. Subsequently, the specified concentrations of *R. mucilaginosa* were added to the ground basal feed accordingly. All mixtures were blended uniformly for 15 min using a drum mixer. Sterile distilled water was then added to the mixtures at a ratio of 40% (w/w) to form dough-like feed. The moistened mixtures were extruded through a pellet mill to produce pellets with diameters ranging from 1.00 to 1.50 mm. The fabricated feed pellets were dried in the air at 30°C until their moisture level reached below 10 g per 100 g. Subsequently, the dried pellets were placed in airtight bags and kept at −20°C for later utilization. A new supply of feed was produced every week. The specific ingredients of the experimental diets are outlined in Table 1.

The viable count of *R. mucilaginosa* in the prepared feed was assessed by employing the plate counting technique [15]. A stock solution was prepared by vortexing 1 g of feed sample in 9 mL of sterilized normal saline. Serial dilutions ranging from 10^3^ to 10^8^ were prepared from the stock solution. For every dilution, 0.1 mL was plated onto sterile nutrient agar (NA) plates, with three replicates for each dilution level. The plates were then incubated anaerobically at 35°C for 48 h. After incubation, colonies were counted and randomly selected for identification to confirm their identity as *R. mucilaginosa*. The number of *R. mucilaginosa* colonies on each plate was calculated. Following colony counting, additional colonies were randomly selected for further identification and isolation of *R. mucilaginosa* strains. The calculation formula is as follows:

  $$\begin{array}{c} \text{The number of viable }R.\text{mucilaginosa}=B\times \frac{C}{10}\times f. \end{array}$$

In the above formula, *B* is the total number of plate colonies on NA medium, *C* is the number of *R. mucilaginosa* colonies identified from 10 colonies, and *f* is the dilution multiple.

According to the NA plate count, no viable *R. mucilaginosa* was detected in the control group diet. In contrast, the viable counts of *R. mucilaginosa* in the experimental diets supplemented with 0.1, 1.0, and 10.0 g/kg were 0.89 × 10^6^, 0.87 × 10^7^, and 0.92 × 10^8^ CFU/g, respectively.

### 2.2. Experimental Animals and Feeding Management

The red claw crayfish utilized in the experiment were obtained from the South Propagation Base of the Guangxi Academy of Fishery Sciences, located in Nanning, China. The initial body weight is 0.13 ± 0.06 g, and the initial body length is 0.58 ± 0.02 cm. All red claw crayfish experiments were conducted in accordance with the guidelines of Guangxi Minzu University, Nanning, China (Approval Number: GXUN 2023-018). Before the culture experiment, red claw crayfish were acclimated in the laboratory's water recirculation system for 7 days. After the acclimation period, young crayfish exhibiting consistent body morphology, absence of limb abnormalities, a healthy appearance, strong vitality, and currently in the intermolt phase were chosen for the subsequent cultivation experiment.

Based on varying concentrations of *R. mucilaginosa* diet, the experiment was designed with four groups: a control group (0 g/kg), a low-dose group (0.1 g/kg), a medium-dose group (1.0 g/kg), and a high-dose group (10.0 g/kg). Each group had three replicates, resulting in a total of 12 aquaculture tanks. Each tank contained 50 shrimp, for a total of 600 shrimp across all tanks. The experimental rearing tanks measured 1 × 2 × 1 m (L × W × H) with a water depth of 0.70 m. Each tank was equipped with 16 PVC pipes (0.75 m diameter, 0.40 m length) as shelters. Aeration stones provided continuous aeration and oxygenation, while aquatic plants were introduced to simulate a natural ecological environment, thereby mitigating stress responses and enhancing dissolved oxygen levels for the crayfish. The environmental conditions were carefully regulated, with the water temperature kept between 26–28°C and the dissolved oxygen levels ranging from 4.7 to 6.2 mg/L, ammonia nitrogen at 0–0.25 ppm, nitrite at 0.01 ppm, nitrate at 0–10 ppm, pH at 7.6–7.8, and natural light (~10 h light: 14 h dark).

Following the formal rearing period, the growth condition of each crayfish in every tank was monitored and documented before each feeding session, and any instances of mortality were recorded. According to the methods of Ren et al. [20], the daily feeding quantity was roughly equivalent to 5% of the crayfish's body weight, with modifications implemented according to actual feeding behaviors. Feedings took place twice a day, at 8:30 in the morning and 6:30 in the evening. Each feeding session consisted of two rounds, with a 30-min interval between them. Every morning after feeding, crayfish excrement was promptly removed, and one-third of the water volume in the rearing tanks was exchanged. The breeding trial lasted for 56 days.

### 2.3. Sampling

At the end of the culture experiment (56 days), the red claw crayfish was starved for 24 h, and then the red claw crayfish was randomly sampled. The 36 red-claw crayfish were randomly sampled in each group (12 red-claw crayfish were extracted from each aquaculture tank). After being rinsed with sterile saline, each crayfish was anesthetized in the ice-water mixture (0°C) for 10 min. According to the methods of Zhang et al. [21], then their body weight was measured individually to calculate the weight gain rate (WGR), specific growth rate (SGR), and feed conversion rate (FCR). The WGR, SGR, and FCR of red claw crayfish were calculated using the following formula:

$\text{WGR }\left(\text{\%}\right)=100\times \frac{\text{final body weight }\left(g\right)-\text{initial body weight }\left(g\right)}{\text{initial body weight }\left(g\right)},$

$\text{SGR }\left(\text{\%}/d\right)=100\times \frac{\left[\ln\left(\text{final body weight}\right)\left(g\right)\right]-\;\left[\ln\left(\text{initial body weight}\right)\left(g\right)\right]}{\text{days}},$

$\text{FCR }=\frac{\text{total feed intake }\left(g\right)}{\text{final body weight }\left(g\right)-\text{initial body weight }\left(g\right)}.$

After the growth performance indicators are calculated, transfer the crayfish to a clean, sterile dissection table. In a sterile environment, the red claw crayfish were carefully dissected, and the hepatopancreas was removed, immediately frozen in liquid nitrogen, and preserved at −80°C.

### 2.4. Determination of Biochemical Parameters and Antioxidant Enzyme Activities

According to the methods of Liu et al. [15], for the measurement of biochemical parameters and antioxidant enzyme activities in the hepatopancreas of red claw crayfish, the samples were first thawed, rinsed with sterile saline, and dried using filter paper. Precisely 1 g of hepatopancreas tissue was weighed and transferred into a 50 mL sterile, enzyme-free centrifuge tube, followed by the addition of nine volumes of physiological saline. The sample was mechanically homogenized on ice and subsequently centrifuged at 1000 × *g* for 10 min at 4°C. The resulting supernatant was collected and diluted with physiological saline to prepare a 1% homogenate for analysis.

The activities of alkaline phosphatase (AKP), acid phosphatase (ACP), superoxide dismutase (SOD), catalase (CAT), glutathione peroxidase (GPX), glutathione S-transferase (GST), and total AOC (T-AOC) and the malondialdehyde (MDA) content in the hepatopancreas were measured using an ELISA analyzer (RT-6100, Rayto, Shenzhen, China) and assay kits. The measurements followed the instructions provided by Nanjing Jiancheng Bioengineering Institute (Nanjing, China). Detailed protocols can be accessed at http://www.njjcbio.com/ (accessed on 8 March 2025).

The activities of AKP and ACP in the hepatopancreas were measured using a micro-enzyme-linked immunosorbent assay. The enzyme activities are expressed in King units per gram of protein (King unit/gprot). One King unit of AKP is defined as the amount of enzyme that produces 1 mg of phenol from the substrate in 15 min at 37°C, while one King unit of ACP is defined as the amount of enzyme that produces 1 mg of phenol from the substrate in 30 min at 37°C. The activity of SOD in the hepatopancreas was measured using the WST-1 method. One unit of SOD activity (U/mgprot) was defined as the amount of enzyme required to achieve 50% inhibition of superoxide radicals in a 1 mL reaction mixture containing 1 mg of tissue protein. The CAT activity in the hepatopancreas was determined by the ammonium molybdate method. One unit of CAT activity (U/mgprot) was defined as the amount of enzyme that decomposes 1 μmol of H_2_O_2_ per second per milligram of tissue protein. The activities of GPX and GST in the hepatopancreas were measured using a colorimetric method. The enzyme activity units were defined as follows: for GPX, one unit (U/mg protein) was defined as the amount of enzyme that caused a reduction of 1 μmol GSH per minute per milligram of tissue protein at 37°C. For GST, one unit (U/mg protein) was defined as the amount of enzyme that caused a reduction of 1 μmol GSH per minute per milligram of tissue protein at 37°C. The T-AOC (mmol/mg prot) in the hepatopancreas was assessed using the ABTS method. The MDA (nmol/mg prot) content in the hepatopancreas was measured using the thiobarbituric acid (TBA) assay.

### 2.5. Determination of Relative Expression Levels of Genes

According to the methods of Zhang et al. [2], the real-time quantitative polymerase chain reaction (RT-qPCR) was employed to determine the relative expression levels of tumor necrosis factor receptor-associated factor 6 (*traf6*), *akirin*, immune deficiency homolog (*imd*), interferon regulatory factor 4 (*irf4*), Toll-like receptor (TLR) 6 (*tlr6*), TLR 2 (*tlr2*), tumor necrosis factor α (*tnf-α*), interleukin-1β (*il-1β*), transforming growth factor-β1 (*tgf-β1*), Janus kinase (*jak*), signal transducer activator of transcription (*stat*), and β*-actin* in the hepatopancreas of red claw crayfish. β*-actin* was selected as the nonregulated internal reference gene. The forward and reverse primers for RT-qPCR were designed using Primer Premier 6.0 software, based on the mRNA sequences of red claw crayfish retrieved from the NCBI database. The synthesis of these primers was carried out by Shanghai Sangon Bioengineering Technology Co., Ltd. (Shanghai, China). For more detailed information regarding the primers, please refer to Table 2.

The brief steps of the RT-qPCR method are as follows: The hepatopancreas tissue of red claw crayfish was homogenized with sterile physiological saline at a 1:10 (w/v) ratio and centrifuged at 1000 g for 10 min at 4°C to obtain the supernatant for RNA extraction. Total RNA was extracted using the Takara MiniBEST Universal RNA Extraction Kit (Takara Biomedical Technology, Beijing, China), with concentration and purity measured by a NanoDrop-2000 spectrophotometer. RNA samples meeting the quality criteria (30–1000 ng/μL concentration and A260/A280 ratio of 1.9–2.1) were subjected to integrity verification through 1% agarose TAE gel electrophoresis using GelRed nucleic acid stain (UVP, Upland, CA, USA), where intact RNA displayed three distinct bands (28, 18, and 5S) with the 28S band intensity being more than twice that of 18S. cDNA was synthesized from 1000 ng total RNA using PrimeScript RT Master Mix (Takara) in a 20 μL reaction system containing 10 μL 2× Taqman PCR mix, 1 μL each of forward and reverse primers, RNA template, and DEPC-treated ddH_2_O, under the following thermal conditions: 30°C for 10 min, 42°C for 15 min, 95°C for 5 min, and 5°C for 5 min. RT-qPCR was performed using the Q2000B Real-Time PCR System (LongGene) with TB Green Premix Ex Taq II (Tli RNaseH Plus, Takara) in a 20 μL reaction volume containing 2 μL cDNA, 10 μL 2× TB Green Premix, 6.4 μL DEPC water, and 0.8 μL each of forward and reverse primers. The RT-qPCR protocol consisted of initial denaturation at 95°C for 30 s, followed by 40 cycles of 95°C for 5 s and 60°C for 20 s. All procedures were carried out under RNase-free conditions to ensure RNA integrity throughout the experimental process.

### 2.6. Data Calculation and Statistics Analysis

In this study, all data were initially entered and sorted using Microsoft Excel 2016. Subsequently, one-way ANOVA was conducted using SPSS Statistics 25.0 to analyze the results data, followed by the creation of relevant charts. To assess significant differences between groups, the least significant difference (LSD) test was employed. GraphPad Prism 9 was utilized for generating the charts. The results of the significance tests are presented as “mean ± standard error” (mean ± SE). Different letters denote statistically significant differences (*p* < 0.05) between groups. Additionally, the relative expression levels of *traf6*, *akirin*, *imd*, *irf4*, *tlr6*, *tlr2*, *tnf-α*, *il-1β*, *tgf-β1*, *jak*, and *stat* genes from fluorescence quantification analysis were calculated using the 2^−ΔΔCT^ method [25].

## 3. Results

### 3.1. Effects of *Rhodotorula mucilaginosa* on the Growth Performance of Red Claw Crayfish

**Figure 1:**
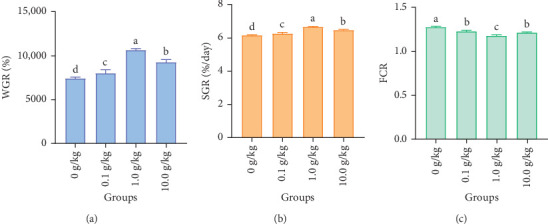
The effects of *Rhodotorula mucilaginosa* on the weight gain rate (WGR; A), specific growth rate (SGR; B), and feed conversion rate (FCR; C) of red claw crayfish. All the data are presented as mean ± SE (*n* = 3). Within the same figure, different superscript letters indicate significant differences between the values (*p* < 0.05).

After 56 days of feeding red claw crayfish with varying levels of *R. mucilaginosa* in their diets, the SGR and WGR in the 0.1, 1.0, and 10.0 g/kg groups were significantly higher than those in the control group (*p* < 0.05). Notably, the 1.0 g/kg group exhibited significantly higher SGR and WGR compared to the other treatment groups (*p* < 0.05), as shown in Figure 1.

After 56 days of feeding red claw crayfish with varying levels of *R. mucilaginosa* in their diets, the FCR in the 0.1, 1.0, and 10.0 g/kg groups was significantly lower than that in the control group (*p* < 0.05). Notably, the 1.0 g/kg group exhibited significantly lower FCR compared to the other treatment groups (*p* < 0.05), as shown in Figure 1.

### 3.2. Effects of *Rhodotorula mucilaginosa* on the Immune and Antioxidant Enzyme Activities in the Hepatopancreas of Red Claw Crayfish

After 56 days of feeding red claw crayfish with varying levels of *R. mucilaginosa* in their diets, the activities (contents) of SOD, CAT, GPX, GST, ACP, and T-AOC in the hepatopancreas in the 0.1, 1.0, and 10.0 g/kg groups were significantly higher than those in the control group (*p* < 0.05). The activity of AKP in the hepatopancreas in the 1.0 g/kg group was significantly higher than that in the control group (*p* < 0.05). However, no significant differences in AKP activity were observed between the 0.1 and 10.0 g/kg groups and the control group (*p* > 0.05). The MDA content in the hepatopancreas in the 0.1 and 1.0 g/kg groups was significantly lower than that in the control group (*p* < 0.05). However, there was no significant difference in MDA content between the 10.0 g/kg group and the control group (*p* > 0.05), as shown in Table 3.

### 3.3. Effects of *Rhodotorula mucilaginosa* on the Relative Expression Levels of Genes in the Hepatopancreas of Red Claw Crayfish

**Figure 2:**
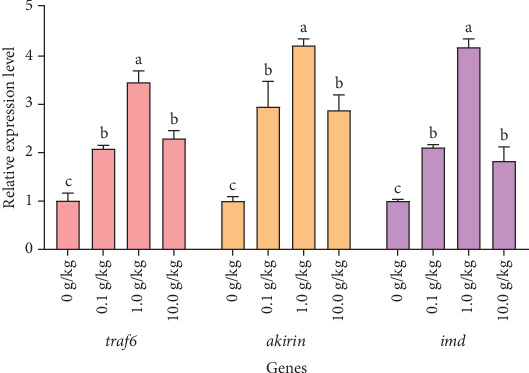
The effects of *Rhodotorula mucilaginosa* on the relative expression levels of tumor necrosis factor receptor-associated factor 6 (*traf6*), *akirin*, and immune deficiency homolog (*imd*) genes in the hepatopancreas of red claw crayfish. All the data are presented as mean ± SE (*n* = 3). Within the same figure, different superscript letters indicate significant differences between the values (*p* < 0.05).

After 56 days of feeding red claw crayfish with varying levels of *R. mucilaginosa* in their diets, the relative expression levels of *traf6*, *akirin*, and *imd* genes in the hepatopancreas in the 0.1, 1.0, and 10.0 g/kg groups were significantly higher than those in the control group (*p* < 0.05). Notably, the 1.0 g/kg group exhibited significantly higher relative expression levels of *traf6*, *akirin*, and *imd* genes compared to the other treatment groups (*p* < 0.05), as shown in Figure 2.

**Figure 3:**
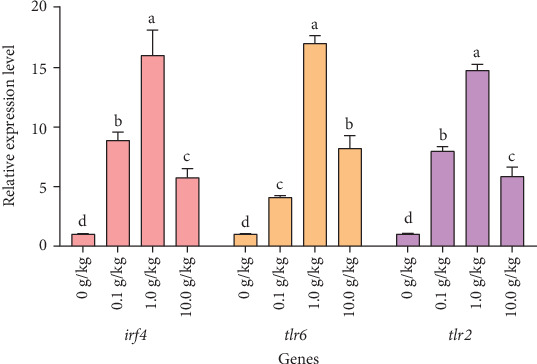
The effects of *Rhodotorula mucilaginosa* on the relative expression levels of interferon regulatory factor 4 (*irf4*), Toll-like receptor 6 (*tlr6*), and Toll-like receptor 2 (*tlr2*) genes in the hepatopancreas of red claw crayfish. All the data are presented as mean ± SE (*n* = 3). Within the same figure, different superscript letters indicate significant differences between the values (*p* < 0.05).

After 56 days of feeding red claw crayfish with varying levels of *R. mucilaginosa* in their diets, the relative expression levels of *irf4*, *tlr6*, and *tlr2* genes in the hepatopancreas in the 0.1, 1.0, and 10.0 g/kg groups were significantly higher than those in the control group (*p* < 0.05). Notably, the 1.0 g/kg group exhibited significantly higher relative expression levels of *irf4*, *tlr6*, and *tlr2* genes compared to the other treatment groups (*p* < 0.05), as shown in Figure 3.

**Figure 4:**
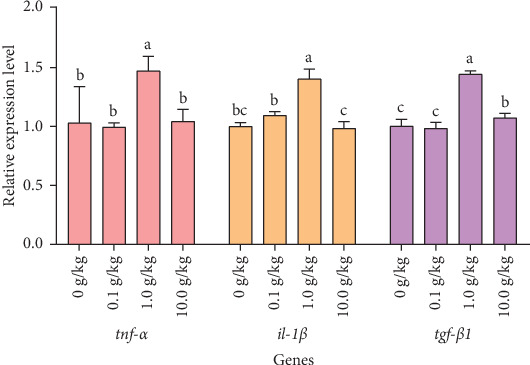
The effects of *Rhodotorula mucilaginosa* on the relative expression levels of tumor necrosis factor α (*tnf-α*), interleukin-1β (*il-1β*), transforming growth factor-β1 (*tgf-β1*) genes in the hepatopancreas of red claw crayfish. All the data are presented as mean ± SE (*n* = 3). Within the same figure, different superscript letters indicate significant differences between the values (*p* < 0.05).

After 56 days of feeding red claw crayfish with varying levels of *R. mucilaginosa* in their diets, the relative expression levels of *tnf-α* and *il-1β* genes in the hepatopancreas in the 1.0 g/kg group were significantly higher than those in the control group (*p* < 0.05). However, there was no significant difference in the relative expression levels of *tnf-α* and *il-1β* genes between the 0.1 and 10.0 g/kg groups and the control group (*p* > 0.05). After 56 days of feeding red claw crayfish with varying levels of *R. mucilaginosa* in their diets, the relative expression level of *the tgf-β1* gene in the hepatopancreas in the 1.0 and 10.0 g/kg groups was significantly higher than that in the control group (*p* < 0.05). However, there was no significant difference in the relative expression level of the *tgf-β1* gene between the 0.1 g/kg group and the control group (*p* > 0.05), as shown in Figure 4.

**Figure 5:**
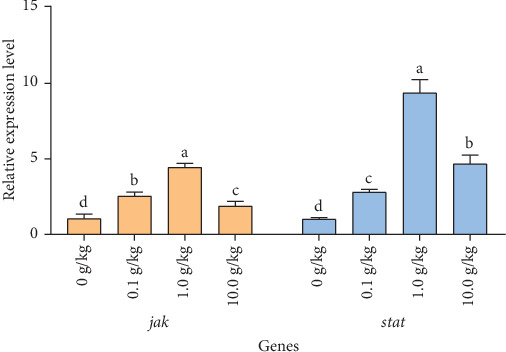
The effects of *Rhodotorula mucilaginosa* on the relative expression levels of Janus kinase (*jak*) and signal transducer activator of transcription (*stat*) genes in the hepatopancreas of red claw crayfish. All the data are presented as mean ± SE (*n* = 3). Within the same figure, different superscript letters indicate significant differences between the values (*p* < 0.05).

After 56 days of feeding red claw crayfish with varying levels of *R. mucilaginosa* in their diets, the relative expression levels of *jak* and *stat* genes in the hepatopancreas in the 0.1, 1.0, and 10.0 g/kg groups were significantly higher than those in the control group (*p* < 0.05). Notably, the 1.0 g/kg group exhibited significantly higher relative expression levels of *jak* and *stat* genes compared to the other treatment groups (*p* < 0.05), as shown in Figure 5.

## 4. Discussion

### 4.1. Effects of *Rhodotorula mucilaginosa* on the Growth Performance of Red Claw Crayfish

SGR, WGR, and FCR are critical metrics for evaluating the growth performance of aquatic animals, directly reflecting their growth efficiency and health status [26]. This research showed that adding *R. mucilaginosa* to the diet greatly improved the WGR and SGR of red claw crayfish and decreased the FCR. Notably, the most pronounced growth promotion was observed when the *R. mucilaginosa* concentration in the diet reached 1.0 g/kg. This phenomenon can be ascribed to the comprehensive nutrient composition of yeast, which comprises amino acids, fatty acids, vitamins, minerals, and substances that regulate immune responses [27, 28]. For instance, lysine, leucine, and arginine in yeast have been shown to attract aquatic animals and promote their growth and development [12]. Furthermore, the β-glucan and mannan oligosaccharides present in the yeast cell wall improve nutrient absorption, which in turn supports increased growth [29]. Consistent with these findings, Yang et al. [19] observed significant enhancements in WGR, SGR, and survival rate of whiteleg shrimp when fed diets supplemented with 10^8^ CFU/g of marine red yeast. Furthermore, this study revealed a dose-dependent response in red claw crayfish, where WGR and SGR initially increased but subsequently declined with increasing dietary levels of *R. mucilaginosa*. This parabolic growth response suggests that excessive yeast supplementation may exert negative effects on growth performance, ultimately leading to reduced WGR and SGR. When the addition of *R. mucilaginosa* reached 10.0 g/kg, it significantly exceeded the optimal dosage, leading to excessive and sustained immune activation. This process consumed a substantial amount of energy and protein resources that should have been allocated for growth. Furthermore, over-supplementation with probiotics can disrupt the normal intestinal microbiota, potentially causing dysbiosis and a decline in digestive and absorptive functions. Additionally, the accumulation of trace metabolic byproducts may exert adverse effects [30, 31]. A parallel phenomenon was observed in juvenile tilapia, where dietary supplementation with *R. mucilaginosa* enhanced WGR, SGR, and FCR, with optimal inclusion levels ranging between 0.53% and 0.60%. Beyond this threshold, excessive yeast supplementation resulted in diminished growth performance in tilapia juveniles [15].

### 4.2. Effects of *Rhodotorula mucilaginosa* on the Immune and Antioxidant Enzyme Activities of Red Claw Crayfish

SOD, CAT, GPX, GST, T-AOC, and MDA play essential roles as key markers for evaluating the AOC and oxidative damage in living organisms [32]. This study demonstrated that supplementation with *R. mucilaginosa* significantly enhanced the activities of SOD, CAT, GSH-PX, GST, and T-AOC in the hepatopancreas of red claw crayfish while markedly reducing MDA levels. These results indicate that *R. mucilaginosa* can enhance the antioxidant and immune functions of red claw crayfish. The mechanisms may be attributed to the following factors: First, carotenoids in yeast hydrolysate effectively scavenge oxygen free radicals [33], thereby mitigating cellular oxidative damage and modulating the activities of SOD and CAT [34]. Second, astaxanthin in *R. mucilaginosa* helps maintain cellular redox balance and reduces ROS levels, indirectly influencing the activities of GPX and GST [35]. Third, yeast cell wall polysaccharides, including β-glucan and mannan, can form complexes with Fe^2+^. This interaction suppresses the generation of ·OH radicals and interrupts lipid peroxidation chain reactions, leading to a reduction in MDA levels [36]. Comparable research has indicated that *Rhodotorula paludigena* can boost the antioxidant and immune capabilities of whiteleg shrimp [37].

ACP is implicated in apoptosis and immune responses, whereas AKP is involved in digestion and immune functions in aquatic animals [38]. In the present research, a notable rise in the activities of both ACP and AKP was detected. This increase could potentially be ascribed to the β-glucan derived from the cell wall of *R. mucilaginosa*. The β-glucan interacts with β-glucan-binding protein-high-density lipoprotein, thereby triggering the prophenoloxidase system. Consequently, this activation enhances the activities of immune-related enzymes [39], thereby promoting increased ACP and AKP enzyme activities. Similar findings were reported in shrimp (*Penaeus vannamei*), where *R. mucilaginosa* was shown to enhance immune enzyme activities and thereby improve host immune responses [40].

### 4.3. Effects of *Rhodotorula mucilaginosa* on the Relative Expression Levels of Genes of Red Claw Crayfish

Crustaceans possess a relatively straightforward immune system that mainly relies on innate immunity to defend against pathogen intrusion. Among the key mechanisms, the Toll and IMD pathways play essential roles in triggering immune genes when faced with pathogen threats [41]. TLR2 and TLR6 are key members of the TLR family, primarily responsible for recognizing pathogen-associated molecular patterns (PAMPs), such as bacterial lipopolysaccharides and fungal β-glucans [42, 43]. In the Toll signaling pathway, TLR2/6 recognizes pathogens by forming heterodimers [44] and recruits the adaptor protein MyD88, subsequently activating TRAF6. TRAF6 then activates TAK1 through K63-linked ubiquitination, ultimately leading to nuclear translocation of NF-κB and expression of antimicrobial peptides (e.g., crustin and lysozyme) [45]. This research discovered that supplementing the diet with *R. mucilaginosa* considerably increased the expression levels of the *tlr2* and *tlr6* genes in the hepatopancreas of red claw crayfish. This upregulation suggests that β-glucans and other components in the red yeast can be recognized by TLR2/6, activating downstream signaling pathways and inducing antimicrobial peptide expression, thereby enhancing immune defense [46]. The increased expression of TLR2/6 not only improves pathogen recognition in crayfish but also systematically clears pathogens through Toll pathway activation, generating immune responses [47]. Similar findings were reported in kuruma shrimp (*Marsupenaeus japonicus*), where upregulated *tlr1/2* expression was closely associated with antiviral responses [48].

The protein encoded by IMD acts as the central element in the IMD signaling pathway, triggering innate immune responses by identifying PAMPs [49]. In the IMD signaling pathway, the protein IMD triggers TAK1 and the IKK complex through adaptor proteins FADD and DREDD. This process subsequently results in the activation of the NF-κB-like transcription factor known as Relish. After relocating to the nucleus, Relish promotes the expression of antimicrobial peptides, which strengthens the host's ability to combat bacterial infections [50]. Our research showed that adding *R. mucilaginosa* to the diet led to a significant increase in *imd* gene expression within the hepatopancreas of red claw crayfish. This pattern of enhancement suggests that *R. mucilaginosa* is capable of strengthening the innate immune response in crayfish by activating the IMD pathway [51]. Activation of the IMD pathway not only facilitates pathogen clearance but also strengthens host immune defense mechanisms by inducing antimicrobial peptide production [52]. These findings are corroborated by parallel research in oriental river prawn (*Macrobrachium nipponense*), where dietary supplementation with *Bacillus coagulans* was shown to significantly upregulate *imd* expression [53].

The protein encoded by *traf6*, serving as a crucial adaptor molecule in both Toll and IMD signaling pathways, exerts its immunomodulatory function by initiating ubiquitination cascades that activate TAK1 and IKK complexes, thereby stimulating NF-κB and MAPK pathways which ultimately induce NF-κB nuclear translocation and antimicrobial peptide expression to regulate immune responses and inflammatory cytokine production [45], while our study demonstrates that *R. mucilaginosa* significantly upregulates *traf6* expression levels, indicating its capacity to enhance innate immunity in red claw crayfish through dual activation of Toll and IMD pathways while maintaining immune homeostasis via inflammatory cytokine modulation [54], a regulatory mechanism corroborated by similar *traf6* upregulation observed in Chinese mitten crab (*Eriocheir sinensis*) [55].

The transcription factors *irf4* and *akirin*, known as key regulatory components in both Toll and IMD signaling pathways [56, 57], exhibit distinct but complementary immunomodulatory functions, where *irf4* mediates TLR signal-dependent regulation of NF-κB-driven gene expression to orchestrate inflammatory responses and immune cell differentiation [58], while *akirin* interacts with the Bap60 subunit and 14-3-3 proteins to modulate NF-κB activity while also forming complexes with Relish to negatively regulate the IMD pathway and suppress target antimicrobial peptide expression [59], with our study demonstrating significant upregulation of both *irf4* and *akirin* expression following *R. mucilaginosa* supplementation, indicating this yeast enhances red claw crayfish immunity through coordinated NF-κB pathway modulation, a conclusion consistent with Chen and Wang's [22] findings during *Vibrio parahaemolyticus* challenge experiments in the same species.

IL-1β and TNF-α belong to the proinflammatory cytokine group. These molecules can stimulate various cells to generate inflammatory mediators and are essential in regulating the inflammatory response [60]. TGF-β1 is a multifunctional cytokine that not only inhibits inflammation but also promotes the apoptosis of inflammatory cells [60]. This research found that *R. mucilaginosa* enhanced the expression of *tnf-α*, *il-1β*, and *tgf-β1* genes in the hepatopancreas of red claw crayfish. The upregulation of *tnf-α* and *il-1β* expressions may be attributed to *R. mucilaginosa* activating TLR2 to recognize foreign antigens, thereby promoting inflammatory signal transduction and enhancing phagocyte function [61, 62]. The increased expression of anti-inflammatory factors such as *tgf-β1* could be a result of immune regulation within the body to prevent excessive activation of proinflammatory factors [60]. Comparable immunomodulatory effects were also noted in marron (*Cherax cainii*). The addition of *Lactobacillus acidophilus* and *L. plantarum* to the diet led to a notable increase in the expression of the proinflammatory cytokines *tnf-α* and *il-1β*. This indicates that probiotic-driven immune activation mechanisms are preserved across different species within the *Cherax* genus [63].

The JAK/STAT signaling pathway, which is also referred to as the cytokine-induced signaling pathway, facilitates the activation of neutrophils and macrophages. It additionally modulates inflammatory reactions and tissue repair, and it is essential in the immune responses of crustaceans [64]. Its core components include cytokine receptors, JAK tyrosine kinases, and STAT transcription factors [41]. When a cytokine signal is detected by its receptor, the JAK tyrosine kinase undergoes phosphorylation. This process triggers the activation and subsequent movement of STAT proteins into the nucleus, where they control the expression of antibacterial peptides [65]. In this research, it was noted that incorporating *R. mucilaginosa* into the diet of red claw crayfish markedly increased the expression levels of *jak* and *stat* genes within the hepatopancreas. This upregulation may represent an adaptive response of the crayfish's immune system to external stimuli. The JAK-STAT signaling pathway is central to immune regulation, promoting the activation, proliferation, and differentiation of immune cells and modulating cytokine production [66]. Consistent with these findings, Sriphuttha et al. [37] demonstrated that *Rhodotorula paludigena* CM33 could activate the JAK-STAT signaling pathways in *Litopenaeus vannamei*, thereby enhancing shrimp immunity.

## 5. Conclusion

In summary, *R. mucilaginosa* markedly promoted growth, boosted the antioxidant capabilities, and strengthened the immune responses of red claw crayfish while activating the Toll/Imd and JAK-STAT signaling pathways. In this experimental context, the ideal addition level of *R. mucilaginosa* was determined to be 1.0 g/kg. Future research will refine this dosage by incorporating a narrower gradient (e.g., 0.8 and 1.2 g/kg) to determine the most effective addition amount for practical application. These findings will provide a solid scientific foundation for developing efficient compound probiotic feed specifically tailored for red claw crayfish.

## Figures and Tables

**Table 1:** The composition of the experimental diets for red claw crayfish (g/kg of dried diet).

Ingredients	*R. mucilaginosa* levels (g/kg)			
	0	0.1	1.0	10.0
*R. mucilaginosa*	0	0.10	1.00	10.00
Fish meal	485.00	485.00	485.00	485.00
Soybean meal	119.30	119.20	118.30	109.30
Sorghum flour	106.50	106.50	106.50	106.50
Wheat flour	139.00	139.00	139.00	139.00
Corn flour	45.00	45.00	45.00	45.00
Soy lecithin	10.00	10.00	10.00	10.00
Fish oil	15.20	15.20	15.20	15.20
Gelatin	20.00	20.00	20.00	20.00
Calcium carbonate	10.00	10.00	10.00	10.00
Choline chloride	5.00	5.00	5.00	5.00
Mineral premixes^a^	20.00	20.00	20.00	20.00
Vitamin premixes^b^	20.00	20.00	20.00	20.00
Vitamin C	5.00	5.00	5.00	5.00
Proximal composition (% dry matter)				
Dry material	92.56	92.56	92.56	92.56
Ash	7.76	7.76	7.76	7.76
Ethereal extract	7.40	7.40	7.40	7.40
Crude protein	35.20	35.20	35.20	35.20
Crude lipid	7.84	7.84	7.84	7.84
Fiber	3.43	3.43	3.43	3.43

^a^Mineral premixes (mg/kg): KCl, 0.5; MgSO_4_·7H_2_O, 0.5; ZnSO_4_·7H_2_O, 0.09; MnCl_2_·4H_2_O, 0.0234; CuSO_4_·5H_2_O, 0.005; KI, 0.005; CoCl_2_·2H_2_O, 0.0025; Na_2_HPO_4_, 2.37.

^b^Vitamin premixes (mg/kg): vitamin B_12_, 0.02; vitamin A acetate, 5000 IU; vitamin D_3_, 4000 IU; α-tocopherol acetate, 100 IU; menadione, 5; thiamin HCl, 60; riboflavin, 25; pyridoxine HCl, 50; folic acid, 10; dl-capantothenic acid, 75; nicotinic acid, 5; biotin, 1; inositol, 5.

**Table 2:** Primer sequences for RT-qPCR in red claw crayfish.

Gene	Primer sequence (5′→3′)	Amplicon size (bp)	Tm (°C)	Gene bank
*β-actin*	F: CGCCTGTCCGCTGGAATAAT	135	60	XM_053800817.1
	R: ACGATGGAAGGGAAGACAGC			

*traf6*	F: GTGCCACAGTCCACCATTCT	262	60	XM_053772658.1
	R: TACCTCTGGCCGCATGAAAG			

*akirin*	F: ACGCCGCAAGATATTACAGTGTGG	112	60	XM_053784413.1
	R: TGATGGTGAGGTAGGACAGACAGG			

*imd*	F: CATACCTCCCCGTCTGTGTCA	[22]	60	[22]
	R: CCATCTAACCCACCTGCTGTC			

*irf4*	F: CAGCGAAGTGTTCCGAGTTCCC	[23]	60	[23]
	R: TATGCCTCCTCCCGTGTGTTCTC			

*tlr6*	F: CTACAGTGCCAATGATGCTACCTAC	105	60	XM_053797426.1
	R: TCGCTGAAGTCTCTGGAGTGAAG			

*tlr2*	F: CTCGGACAAGGAGCGGTTAGTTTC	131	60	XM_053771523.1
	R: TTCTGATTGATAACCTGCTGGAGTCTG			

*tnf-α*	F: ACAGCATTAGTGAGAGCAGCAATC	123	60	XM_053772658.1
	R: CATTAGGACACATAACTGGTCTGAGG			

*il-1β*	F: ACGGTCACAGCCTCTAATGGTAC	78	60	XM_053781109.1
	R: CTCTCGGTAGTTCGGATTGGTTTG			

*tgf-β1*	F: CTCCAACACCACCTGAAGATAGATTG	98	60	XM_053797306.1
	R: AGTAACAGTGACATAGCAGTAACCATC			

*jak*	F: TGTGAGGCATAACAGTAACGAAGG	[24]	60	[24]
	R: GCCCAAGGAACTCAATGGAATG			

*stat*	F: CAGAAAATGTAGCCCACAGCCAG	[24]	60	[24]
	R: TAAAGCAAGGGGATTATTATTCAGG			

*Note:* F, forward primer; R, reverse primer. imd, immune deficiency homolog. β-actin, nonregulated reference gene.

Abbreviations: il-1β, interleukin-1β; irf4, interferon regulatory factor 4; jak, janus kinase; tgf-β1, transforming growth factor-β1; tlr2, Toll-like receptor 2; tlr6, Toll-like receptor 6; traf6, tumor necrosis factor receptor-associated factor 6; tnf-α, tumor necrosis factor α; stat, signal transducer activator of transcription.

**Table 3:** The effects of *Rhodotorula mucilaginosa* on the immune and antioxidant enzyme activities in the hepatopancreas of red claw crayfish.

Index	*R. mucilaginosa* levels (g/kg)			
	0	0.1	1.0	10.0
SOD (U/mgprot)	46.74 ± 2.06^b^	54.77 ± 1.08^a^	57.78 ± 2.36^a^	57.73 ± 2.90^a^
CAT (U/mgprot)	8.73 ± 0.33^c^	12.05 ± 0.90^b^	16.13 ± 0.42^a^	14.25 ± 0.95^ab^
GPX (U/mgprot)	574.01 ± 23.75^c^	618.64 ± 24.11^b^	683.07 ± 21.09^a^	631.24 ± 20.32^b^
GST (U/mgprot)	16.65 ± 3.17^b^	19.27 ± 2.13^a^	24.33 ± 4.01^a^	27.16 ± 2.02^a^
T-AOC (mmol/mgprot)	0.13 ± 0.01^c^	0.18 ± 0.01^b^	0.24 ± 0.01^a^	0.20 ± 0.01^b^
ACP (King unit/gprot)	278.99 ± 27.67^c^	353.16 ± 43.55^b^	424.36 ± 30.77^a^	332.81 ± 34.23^b^
AKP (King unit/gprot)	152.70 ± 16.52^b^	151.34 ± 14.76^b^	203.27 ± 16.42^a^	164.01 ± 11.91^b^
MDA (nmol/mgprot)	1.50 ± 0.05^a^	1.30 ± 0.04^b^	1.18 ± 0.04^c^	1.45 ± 0.04^a^

*Note:* All the data are presented as mean ± SE (*n* = 3). Within the same row, different superscript letters indicate significant differences between the values (*p* < 0.05).

Abbreviations: ACP, acid phosphatase; AKP, alkaline phosphatase; CAT, catalase; GPX, glutathione peroxidase; GST, glutathione s-transferase; MDA, malondialdehyde; SOD, superoxide dismutase; T-AOC, total antioxidant capacity.

## Data Availability

The data that support the findings of this study are available from the corresponding author upon reasonable request.
